# Does Religiosity Reduce Mortality Risk Over the Second Half of Life Among Southern Californians? A Multidimensional Model Within a Hazard Modeling Framework

**DOI:** 10.1007/s10943-025-02297-4

**Published:** 2025-04-05

**Authors:** Maria Teresa Brown, Wencheng Zhang, Woosang Hwang, Merril Silverstein

**Affiliations:** 1 Aging Studies Institute, Syracuse University, 314 Lyman Hall, Syracuse, NY 13244 USA; 2 School of Social Work, Syracuse University, Syracuse, NY USA; 3 Department of Sociology, Purdue University, West Lafayette, IN USA; 4 Department of Family & Resource Management, Sookmyung Women’s University, Seoul, South Korea; 5 Department of Sociology, Syracuse University, Syracuse, NY USA; 6 Department of Human Development and Family Science, Syracuse University, Syracuse, NY USA

**Keywords:** Religion, Mortality, Latent class analysis, Hazard modeling, Longitudinal data

## Abstract

This study explored the relationship between a multidimensional typology of religiosity and risk of mortality. Using data from middle-aged Southern California respondents in the Longitudinal Study of Generations, we performed latent class analysis to construct a typology of religiosity, which was then used to predict mortality from 1971 to 2020. We identified four religiosity classes: strongly, weakly, privately, and liberally religious. Cox proportional hazard models revealed that privately religious males had greater mortality risk than strongly religious males, even after controlling for the mediators self-rated health and psychological wellbeing. In women, risk of mortality was explained by these mediators rather than by religiosity.

## Introduction

Over the last three decades, researchers have been studying the influence of religion and religiosity on health and mortality. A variety of studies have found that religiosity, whether defined as religious service attendance, religious intensity, or religious beliefs and experiences, is associated with better health outcomes and greater longevity (Dupre et al., 2006; Hummer et al., 1999; McCullough et a., 2000; Musick et al., 2004; Strawbridge et al., 1997). These positive effects of religious involvement have been identified in the United States, Taiwan, Northern Ireland, and China, and various racial/ethnic groups have been found to experience these positive benefits (Zhang, 2008). Strength of religiosity is negatively associated with all-cause mortality as well as several specific causes of mortality (Hummer et al., 1999).

Most studies of the relationship between religiosity and mortality are limited to measures such as religious attitudes and beliefs (Lechler & Sunde, 2020) or religious behaviors like prayer and church attendance (Darviri et al., 2016; Stavrova, 2015). Recent multinational research found consistent positive effects of religiosity on self-rated health, an outcome selected due to its strength as a predictor of mortality (Zimmer et al., 2019); however, only two of three unidimensional religiosity measures were directly related to religious behavior and beliefs. We have extended this line of research by examining multiple dimensions of religiosity, including religious participation, intensity, civic value, and literal beliefs. Using a person-centered approach through latent class analysis, we identified groups of people who share similar patterns across these dimensions. Additionally, we studied time-varying religiosity over a period of up to 26 years and mortality over four decades, which is rarely explored in existing literature, to examine how religiosity in midlife might predict mortality over time.

## Multiple Dimensions of Religiosity

Religiosity encompasses various dimensions, including institutional aspects such as affiliation, behavioral aspects like attendance of services, prayer, and reading sacred texts, and attitudinal aspects such as the salience, value of religion, and religious intensity (Idler et al., 2003; Lee et al., 2018; Lenski, 1961; Pargament et al., 2013; Park, et al., 2013; Zinnbauer et al., 1999). Research studying health outcomes of religion should consider multiple dimensions of religion and spirituality, as each dimension may be linked to health and mortality in different ways (Idler et al., 2003). Recent studies examining the relationship between multiple dimensions of religion and health either examined the effects of each dimension separately or created a composite score (Darviri et al., 2016).

There are several potential advantages to examining multiple dimensions of religiosity using a person-based typological approach. First, this approach allows asymmetrical consideration of different dimensions where a person might have higher score on one dimension and low score on another (Park, et al., 2013). For example, some research has found that a high level of religious intensity combined with a low level of religious attendance may be associated with worse psychological wellbeing, probably because those with higher religious intensity tend to feel more guilt when they have low attendance (Dillon & Wink, 2007). Second, this approach avoids the estimation of higher order interaction terms which could be difficult to interpret. Third, when religious dimensions are highly correlated with each other (Faulkner & de Jong, 1966; Park et al., 2013; Pearce et al., 2017), person-centered analysis has advantages when the dimensions are examined simultaneously. Latent class analysis (LCA) is a person-centered approach which allows identification of unobserved subgroups inferred from participants’ responses to religiosity indicators (Nylund-Gibson & Choi, 2018). LCA allows researchers to identify subgroups based on combinations of a full array of religious characteristics (Bravo et al., 2016). In our application we use this technique to identify groups of people who share similar patterns on various dimensions of religiosity.

Studies using LCA have efficiently identified religiosity groupings based on unique combinations across categorical religious dimensions. For example, Hwang et al. (2024) identified a four-class religiosity typology: strongly religious, weakly religious, personally religious, and doctrinally religious. Personally religious referred to people who were highly religious and spiritual but low in attendance, civic value of religion, and literal beliefs of religion, while doctrinally religious referred to people who were high in civic values and literal beliefs in religion but low in religiosity, spirituality, and service attendance. Grill and colleagues (2020) identified three groups using latent class analysis among persons living with HIV: patients who were traditionally religious, privately religious, and spiritual but not religious. They found that traditionally religious individuals had better mental health, quality of life, and physical health than the other two groups, and patients in the privately religious group had the lowest mental health and quality of life (Grill et al., 2020).

A related technique of latent profile analysis uses continuous measures to identify religious groups. Bravo et al. (2016) identified four underlying groups using latent profile analysis: high religiosity, low religiosity, questioning, and intrinsically motivated. The questioning group was characterized by high questioning in one’s religiosity and low extrinsic and intrinsic religiosity while the intrinsically motivated group had high intrinsic religiosity and low extrinsic and questioning religiosity. Their study found that the intrinsically motivated group had better psychological wellbeing than the other three groups (Bravo, et al., 2016).

These previous studies demonstrate how the combinations of different dimensions of religiosity might be associated with health and wellbeing. However, most studies utilizing latent class analysis have created typologies of religiosity based on one time point, ignoring the fact that people’s religiosity could change over time. The current study addresses this gap by creating a religiosity typology based on up to three waves of data over 26 years.

In the current study, we applied LCA to the multi-wave Longitudinal Study of Generations (LSOG) to examine how membership in multidimensional religious classes in midlife predicted risk of mortality with aging up to four decades later. Prior research using the LSOG has identified typologies of religiosity, and class membership as applied to various outcomes. For example, in a cross-sectional analysis of religiosity class membership in three-generation families, researchers found a heterogeneous mix of classes in each of three generations—the Silent Generation (G2), the Baby Boom generation (G3), and Generation X (G4) (Hwang et al., 2024). Other studies employed latent class analysis of religiosity to explore the relationship between religious class membership and outcomes like filial norms in Baby Boomer and Gen-X participants in early middle age (Hwang et al., 2021), and between transitions in religious class membership over time and psychological wellbeing in Baby Boom and Gen-X participants in later life (Hwang et al., 2022, 2023a, 2023b). To address the issue of temporality, these previous studies demonstrated that membership in strongly religious and weakly religious classes (similar ranking of importance of religious beliefs and religious behaviors) was largely stable across the term of study, while this was not the case in intermediate religiosity classes (differing rankings of importance of religious beliefs versus religious behaviors). Overall, religious class membership followed predictable life span pathways with relatively few deviations from baseline.

### Religiosity and Mortality

A considerable number of studies have shown that a higher level of religious service attendance is associated with lower all-cause mortality in the U.S. (Dupre et al., 2006; Gillum et al., 2008; Hart, 2001; Hill et al., 2005; Hummer et al., 1999; Li et al., 2016; Oman & Reed, 1998; Schnall et al., 2010; VanderWeele et al., 2017), in Europe (Christopoulos, 2023; Darviri, et al., 2016), and in East Asia (Hidajat et al., 2013; Zeng et al., 2011; Zimmer et al., 2020). Meta-analyses examining the literature on this association found that less frequent participation in religious activities was associated with almost 30% increased risk of all-cause mortality (McCullough et al., 2000), higher religiosity with an 18% reduction in mortality risk (Chida et al., 2009), and more frequent service attendance with a 25% reduction in mortality (Powell et al., 2003).

However, some studies indicate that the impact of service attendance may be suppressed by poor health status. For instance, Chida et al. (2009) found that religious service attendance was associated with longevity among healthy individuals but not among those with serious illnesses. Similarly, Helm et al. (2000) reported that private religious activities such as prayer benefited older adults without Activities of Daily Living (ADL) impairments, but not those with ADL impairments. These findings suggest that the protective effects of religious involvement may be more pronounced in preventing disease rather than helping recovery (Buja et al., 2024; Chida et al., 2009; Helm, et al., 2000). In addition, a literature review by Koenig (2015) found that although the majority of studies showed religion/spirituality involvement was associated with lower mortality, few reported higher mortality. Thus, the relationship between religiosity and mortality is complex and requires further examination.

Findings from past research on the relationship between religious intensity and mortality are also mixed. Clark et al. (1999) found that women who were more religious in midlife had lower premature mortality risk, while Kim, Smith, and Kang (2015) reported that intensity of religious affiliation did not significantly influence mortality. A study of Jewish Israeli males found that self-reported religiosity was associated with lower mortality in younger (< 55 years old) participants but higher mortality in older (> = 55 years old) participants (Kraut et al., 2004).

Most studies of religion and mortality have considered religiosity at a baseline measurement period without considering that religious involvement tends to change over the adult life course (Hayward & Krause, 2013b). The current study addressed this gap by updating religiosity assessments for three waves of measurement over 26 years and examining how time-varying membership in religious categories predicted mortality outcomes.

### Psychological and Physiological Mediators

This study focuses on two mediators—psychological wellbeing and self-rated health—that might explain the link between religiosity and longevity. Religious attendance and intrinsic religiosity have been linked to fewer depressive symptoms (Croezen et al., 2015) and stronger belief in God-mediated control has been associated with greater life satisfaction (Krause, 2005). The positive association with psychological wellbeing may be partly due to religion’s social integration function (McCullough et al., 2000). Religious involvement provides tangible and emotional support (Hayward & Krause, 2013a) and fosters a sense of belonging and purpose, ultimately promoting wellbeing (Michaels et al., 2022; Robbins & Francis, 2000). Meanwhile, better psychological wellbeing has been linked to lower mortality risk (Gilman et al., 2017). Based on these prior studies, we examined whether psychological wellbeing played a mediating role in the relationship between religiosity and longevity.

We also examined self-rated health as a mechanism linking religiosity and mortality due to its association with both factors. Studies have shown that individuals who were more religious and who participated in more religious activities tended to report better self-rated health (Doane & Elliott, 2016; Reyes-Ortiz et al., 2019). This may be partially explained by religion’s regulatory function, which offers rules and norms for diet, lifestyle, marriage, childbirth, and family life, thereby influencing health behaviors. For example, research has found associations between religiosity and lower rates of smoking and substance use (Gorsuch, 1995; Kendler et al., 1997), and reduced risk of engaging in risky sexual behaviors (Simons et al., 2009). Healthy lifestyles, in turn, are linked to better self-rated health (Zhang et al., 2020). We focus on self-rated health because it is a comprehensive measure capturing objective and subjective aspects of overall health (Jylha, 2009; Tissue, 1972). Self-rated health is consistently found to be a strong indicator of mortality in numerous studies, underscoring its importance in reflecting underlying health conditions (DeSalvo et al., 2006; Idler & Behyamini, 1997; Jylha, 2009). Given the connections between self-rated health, religiosity, and mortality, we hypothesized that self-rated health would mediate the relationship between religiosity and mortality.

### Moderating Effect of Gender

There are two competing frameworks in terms of the moderating effects of gender on the association between religious involvement and wellbeing (McFarland, 2009). The first framework suggests that women may derive greater health benefits from religious involvement than men due to several factors. First, women tend to build and maintain larger social networks and provide more emotional support to others than men (Kahn et al., 2011; McLaughlin et al., 2010). This may help women build stronger ties in church, which is beneficial for their health and wellbeing. Second, women have been found to be more religious than men and utilize religious coping mechanisms more often than men (Hvidtjorn et al., 2014; McCullough et al., 2000; Schnabel, 2017), suggesting that women may leverage the psychosocial resources of religion more effectively than men. Supporting this, a meta-analysis by Mccullough et al (2000) found a stronger negative association between religious service attendance and mortality among women compared to men, and Strawbridge et al. (1997) showed that the protective effects of frequent religious service attendance were more pronounced for women than for men.

A contrasting perspective suggests that religious involvement may be more beneficial for men than women. This argument posits that traditional gender roles, which often emphasize male self-reliance, may limit men’s access to emotional support outside of religious contexts (Lease et al., 2012). Religious participation can provide a space for men to express vulnerability and seek support, which is crucial for mental health (McFarland, 2009). In contrast, access to diverse social networks both within and outside religious communities, potentially reducing women’s reliance on religion for emotional support. Thus, religious involvement may be particularly critical for men’s longevity due to its positive impact on mental health. Additionally, patriarchal gender norms in society at large are reflected in church settings, with men holding more leadership positions and women clergy over-represented in subordinate roles (Sullins, 2000). Thus the higher status of men in many religious institutions might lead to more mental health benefits for men than for women. Supporting evidence shows that religion involvement is more beneficial for men’s mental health (Maselko & Kubzansky, 2006; McFarland, 2009), cardiovascular health (Tartaro et al., 2005) and self-rated health (Maselko & Kubzansky, 2006) when compared to similar benefits accrued by women. Based on the evidence from both perspectives, we stratified our models to account for the potential role of gender in moderating the association between religious involvement and mortality.

### The Current Study

In the current study, we identified a typology of religiosity among Silent Generation (G2) participants in the 1971, 1988, and 1997 waves of the LSOG, to explore the association between religiosity at baseline in 1971 and risk of mortality as individuals aged for as many as 49 years from baseline. While previous typology studies have explored religious class membership and later life outcomes among Baby Boom and Gen-X participants, the current study is unique in examining whether religious classes among Silent Generation participants predict the risk of mortality over their life course. The Silent Generation of the LSOG sample is better-suited to this analysis than subsequent generations, as the data include measures for multiple dimensions of religiosity at midlife and we were been able to connect to National Death Index (NDI) data for 73% of the G2 sample.

The current study tested three hypotheses: H1: Respondents will belong to one of several discernable classes of religiosity, including strongly and weakly religious and at least one intermediate class of religiosity; H2: Respondents who were strongly religious at baseline will have lower risk of mortality compared to those who were less religious, an association that will differ by gender; and H3: Accounting for health and psychological wellbeing will partially explain the association predicted in H2.

## Method

### Sample

The current study utilized the first wave of the Longitudinal Study of Generations (LSOG), which was collected in 1971. Wave 1 surveyed 2444 respondents within 358 three-generation families, including grandparents (G1), parents (G2), and grandchildren (G3). Respondents were sampled using a multistage stratified random sampling of grandfathers and their spouses in the largest health maintenance organization in southern California. Follow-up surveys were conducted in 1985, 1988, 1991, 1994, 1997, 2000, 2005, and 2016 (for more details, see Bengtson, 2013; Silverstein & Bengtson, 2019). Data were mainly collected through mail-back questionnaires, although some data was collected through telephone interviews, computer-assisted self-administered surveys, and face-to-face interviews. Beginning in 2016, respondents also had the option to respond to web-based questionnaires.

The current study included data from 701 G2 s who participated in the 1971 survey (Wave 1). We excluded 3.71% of the 1971 sample because of missing information on key variables, resulting in a sample size of 675 G2 adult respondents, of whom 305 (45.2%) were male and 370 (54.8%) were female. If available, additional data to update predictors were obtained from the 1988 (Wave 3) and 1997 (Wave 6) surveys. If data were not available in 1988 or 1997, values were carried forward from the last wave in which data was available for that participant. In terms of ascertaining vital status, 491 (72.7%) were confirmed as having died between 1971 and 2020 and 184 were determined to be alive at their final measurement and considered censored observations.

### Measures

#### Mortality

The dependent variable explored in the current study is age at death, which is measured by linking LSOG survey data to mortality data obtained from the National Death Index (NDI) for the years 1979 through 2020 (National Center for Health Statistics, 2020). Matches to the NDI (*n* = 457) were based on respondent’s name, date of birth, gender, and Social Security number. Ascertaining mortality status was based on the matching quality of the records as well as the probabilistic scorings provided by the NDI. We utilized mortality information reported by family members for respondents not matching to the NDI (*n* = 34) and for all respondents who died prior to 1979. In this study, dynamic risk of morality is operationalized using age at death or censoring from 1971 to 2020.

#### Religiosity

Participants’ religiosity was measured based on four dimensions (religious participation, religious intensity, civic value of religion, and literal belief) collected in the 1971 (Wave 1), 1988 (Wave 3), and 1997 (Wave 6) surveys. Participants’ religious participation was measured by one item (“how often do you attend church or religious services these days?”). Response options ranged from (1) *never* to (6) *more than once a week*. We dichotomized responses into low (*never*-*several times a year*) and high (*once a month-more than once a week*) categories.

Participants’ religious intensity was measured by one item (“regardless of whether you actually attend religious services, do you consider yourselves to be religious?”). Response options ranged from (1) *not at all religious* to (4) *very religious*. We dichotomized responses into low (*not at all religious-somewhat religious*) and high (*moderately religious-very religious*) categories. Participants’ civic value of religion were measured by one item (“This country would be better off if religion had a greater influence on daily life”). Response options ranged from (1) *strongly disagree* to (4) *strongly agree*. We dichotomized responses into low (*strongly disagree-disagree*) and high (*agree-strongly agree*) categories.

Participants’ literal belief was measured with two items (“God exists in the form as described in the Bible” and “All people alive today are descendants of Adam and Eve”). Response options ranged from (1) *strongly disagree* to (4) *strongly agree*. We dichotomized responses into low (*strongly disagree-disagree*) and high (*agree-strongly agree*) categories. Participant responses from 1971 were carried forward to 1985 and 1988 if they did not respond to the survey in those waves.

#### Demographic Controls

To account for the influence of demographic confounders, we included controls from the 1971 data for educational attainment (lower than high school = 1, high school = 2, some college = 3, bachelor’s degree or above = 4), and religious denomination, and marital status (married = 1, not married = 0) as a time-varying variable. We stratified our results by gender (female = 1, male = 0).

#### Mediators

*Self-Rated Health.*To better understand the process by which religiosity is related to mortality risk, we included self-rated physical health and psychological wellbeing as potential time-varying mediators, each measured from 1971 through 1997. Physical health was measured as self-rated health. In 1971, respondents were asked whether they would describe their general health condition as excellent (yes, no, or don’t know) or poor (yes, no, or don’t know).

We created the variable of self-rated health in 1971 by combining the questions on excellent health (HEALTH5) and poor health (HEALTH8). See Appendix 1 for the list of the nine general health items in the 1971 LSOG survey. Specifically, self-rated health was coded as 1 when excellent health = yes and poor health = no or don’t know, and self-rated health was coded as 0 when poor health = yes and excellent health = no or don’t know. Respondents were coded as 0.5 if they reported their health as neither excellent nor poor (16.9%) or both excellent and poor (0.3%), or if they answered “no” to poor health and “don’t know” to excellent health (11.4%) or “no” to excellent health and “don’t know” to poor health (1.9%). Finally. respondents who were missing on either question (14.52%) were coded as 0.5.

In the 1988 and 1997 surveys, self-rated health was measured by the question “Compared to people your own age, how would you rate your overall physical health at the present time?” Responses included excellent, good, fair, and poor. We recoded the answers to make them as consistent as possible with the 1971 baseline measure whereby 1 = excellent, 0.75 = good, 0.25 = fair, and 0 = poor. Respondents with missing values in 1988 (21.6%) and 1997 (21.4%) had their health score carried forward from the last wave in which they responded to the self-rated health item. In some cases, that may be the imputed value from 1971, but in other cases the 1985 values may be carried over to 1988 or the 1988, 1991 or 1994 values might be carried over to 1997. A dichotomous control was created to account for participants whose self-rated health measures were missing (1 = yes) in 1971, 1988 or 1997.

*Psychological WellBeing.*We measured respondent psychological wellbeing in 1971, 1988, and 1997 with Bradburn’s Affect Balance Scale(Bradburn, 1969). The Affect Balance Scale in the LSOG survey included 10 items from the Bradburn scale. Respondents were asked about their wellbeing in the “past few weeks” prior to the survey. Five dichotomous positive items included feeling “particularly excited or interested in something” and “things were really going your way.” Five dichotomous negative items included feeling “very lonely or remote from other people” and “depressed or very unhappy.” After reverse coding the negative items, we summed these dichotomous items to create a wellbeing score potentially ranging from 0 to 10, with a higher value representing more positive feelings (Cronbach’s alpha = 0.62). See Appendix 2 for a list of the ten Bradburn Affect Balance Scale items in the LSOG survey. Participants who were missing on Bradburn scores in 1988 (22.2%) or 1997 (20.9%) were assigned values carried forward from previous waves.

### Analytic Strategy

#### Latent Class Analysis

Using data from Wave 1 (1971), Wave 3 (1988), and Wave 6 (1997) we first examined sample characteristics, then performed a latent class analysis to construct a latent class typology of religiosity. Finally, we estimated Cox proportional hazard models to predict years to death along an age gradiant between 1971 and 2020, based on membership in religiosity classes and controlling for baseline age, gender, education, marital status, religious denomination, and health and psychological wellbeing over time.

We addressed the first hypothesis by conducting a latent class analysis incorporating five dichotomized religiosity indicators in the 1971, 1988, and 1997 surveys using Latent Gold 6.0 (Vermunt & Magidson, 2021). We dichotomized all religiosity indicators for the latent class analysis because most variables were skewed and therefore were not able to capture the full range of the scale; in this case dichotomization is recommended (DeCoster et al., 2009; Macia & Wickham, 2019; Vasilenko, 2022). Relatedly, previous studies showed that nominal indicators of religiosity often identify more distinguishable and interpretable classes than ordinal or continuous indicators of religiosity (Hwang, et al., 2023a, 2023b; Lee, et al., 2018; Pearce et al., 2013; Suitor et al., 2018; Vasilenko & Espinosa-Hernandez, 2019). We selected the optimal number of latent classes using Bayesian Information Criterion (BIC), Akaike Information Criterion (AIC), and Sample-Adjusted Bayesian Information Criterion (SABIC). The smallest values of BIC, AIC, and SABIC considered the optimal fitting model (Nylund-Gibson & Choi, 2018). In addition, full information maximum likelihood estimation (FIML) was utilized to account for missing values.

#### Cox Proportional Hazard Models

We addressed the second and third hypotheses using Cox proportional hazard models in Stata 17.0 (Cox, 1972, 1975) to evaluate the relationship between religiosity class membership and age-related mortality risk from 1971 to 2020. Cox models provide a strong basis for predicting mortality with relatively few parametric assumptions, estimating relative risk of mortality associated with static and time-varying predictors and accounting for censored observations for survivors and attritors in the sample. Respondents without a verifiable report of death were treated as censored for the last year in which they were ascertained as being alive by virtue of participating in the survey or from reports of family members. We applied the “last value carried forward” method to determine the values of updated time-varying covariates in assessing mortality risk beyond and between survey years of 1971, 1988, and 1997.

We first assessed the relationship between religiosity and mortality controlling only for demographic variables—age, educational attainment, marital status, and religious denomination—then added self-rated health and psychological wellbeing as mediators. We applied Cox proportional hazard models separately for men and women to account for gender differentials in religiosity and age of death.

## Results

### Sample Description

As presented in Table 1, the majority of the sample were women (54.81%). On average, the G2 s were 43.7 years old in 1971, with men being older than women (45.5 vs. 42.2, respectively). Men (0.78 (0.27), range 0–1, higher = better health) and women (0.71 (0.27)) were both in good health and had a positive attitude (7.02 (1.94) and 6.87 (2.2), respectively, 1–10, higher = more positive). A larger proportion of men in this sample had at least a bachelor’s degree (21.5%), whereas a larger proportion of women in this sample had only a high school diploma or the equivalent (39.5%). In terms of religiosity, the majority of the sample identified as Mainline Protestants (39.3%), followed by Catholics (18.22%) and Evangelical Protestants (14.7%). Another 13.3% of the sample identified as Jewish, 6.7% identified as having no religious denomination, and the remaining 3.3% of the sample was comprised of various other denominations, including Mormons.

**Table 1:** Participant demographic characteristics (*N* = 675), longitudinal study of generations, 1971

	Full sample		Men (*N* = 305)		Women (*N* = 370)		Sig
Characteristic	Mean (SD)	#/%					
Number of deaths (1971–2021)		491		259		232	***
Age, 1971	43.67 (5.28)		45.50 (5.37)		42.24 (4.75)		***
Female, 1971		54.81%					
Education, 1971							***
Less than high school		12.30%		11.80%		12.70%	
High school		31.70%		22.30%		39.46%	
Some college		34.37%		33.44%		35.14%	
Bachelor’s degree or above		21.63%		21.46%		12.70%	
Married, 1971		93.93%		96.72%		91.62%	**
Religiosity continuous membership probability, 1971							
Strongly religious	0.42 (0.42)		0.42 (0.43)		0.42 (0.41)		
Privately religious	0.17 (0.33)		0.20 (0.36)		0.15 (0.31)		
Weakly religious	0.28 (0.35)		0.26 (0.35)		0.29 (0.34)		*
Liberally religious	0.13 (0.28)		0.12 (0.27)		0.14 (0.29)		
Religious denomination, 1971							
Mainline Protestant		39.26%		40.33%		38.38%	
Evangelical Protestant		14.67%		13.77%		15.41%	
Catholic		18.22%		17.70%		18.65%	
Jewish		13.33%		14.43%		12.43%	
Others		3.26%		6.56%		8.92%	
None		6.67%		7.21%		6.22%	
Excellent health, 1971 (0–1, 0 = poor health, 1 = excellent health)	0.74 (0.27)		0.78 (0.27)		0.71 (0.27)		**
Missingness in excellent health		14.52%		13.77%		15.14%	
Bradburn psychological wellbeing, 1971 (1–10, higher = more positive feeling)	6.94 (2.06)		7.02 (1.94)		6.87 (2.15)		

### Latent Class Analysis

Latent class analysis statistics and fit indices are presented in Table 2. The BIC, AIC, and SABIC values suggested that a four-class model was the optimal fitting model in the 1971, 1988, and 1997 surveys. This analysis supports our first hypothesis, that members of the silent generation would belong to one of several classes of religiosity, including strongly and weakly religious and at least one additional intermediate class of religiosity.

**Table 2:** Latent class analysis statistics and fit indices among G2 s, longitudinal study of generations, 1972–1977

	Classes (n)	BIC	AIC	SABIC
1971 Survey (*n* = 701)				
Model 1	1	4229.00	4206.25	4213.13
Model 2	2	3576.01	3525.94	3541.08
Model 3	3	**3541.28**	3463.91	3487.30
**Model 4**	**4**	3543.37	**3438.70**	**3470.34**
Model 5	5	3579.49	3447.51	3487.41
Model 6	6	3618.21	3458.92	3507.08
1988 Survey (*n* = 569)				
Model 1	1	3586.88	3565.16	3571.00
Model 2	2	3068.36	3020.57	3033.44
Model 3	3	3042.85	2969.00	2988.88
**Model 4**	**4**	**3027.97**	**2928.06**	**2954.96**
Model 5	5	3061.69	2935.72	2969.63
Model 6	6	3098.21	2946.18	2987.11
1997 Survey (*n* = 498)				
Model 1	1	3124.07	3103.02	3108.20
Model 2	2	2572.68	2526.36	2537.76
Model 3	3	**2581.20**	2509.62	2527.25
**Model 4**	**4**	2590.82	**2493.97**	**2517.81**
Model 5	5	2622.75	2500.64	2530.70
Model 6	6	2659.11	2511.74	2548.02

Bolded values indicate best fit for each respective statistic. BIC, Bayesian Information Criterion. AIC, Akaike Information Criterion. SABIC, Sample-size Adjusted Bayesian Information Criterion

Item response and latent class probabilities of the four religiosity classes across 1971, 1988, and 1997 surveys are presented in Table 3. We identified the same four latent classes in each of the three waves based on the number of classes of the best fitting model and configural similarities in the conditional probabilities defining the classes across the waves. Results of a chi-square test for differences showed that distribution of these classes was not significantly different across the three waves.

**Table 3:** Item response and latent class probabilities of religiosity latent classes

Item response probabilities	Strongly religious	Weakly religious	Privately religious	Liberally religious
1971 survey (*n* = 701)				
Religious attendance	**0.77**	0.01	0.01	0.29
Religious intensity	**0.96**	0.09	**0.62**	**0.89**
Country would be better off	**0.99**	0.29	**0.85**	**0.62**
God in the Bible	**0.98**	0.01	**0.90**	0.05
Adam and Eve	**0.85**	0.04	**0.68**	0.11
Latent class probabilities	0.42	0.17	0.28	0.13
1988 survey (*n* = 569)				
Religious attendance	**0.75**	0.02	0.10	0.38
Religious intensity	**0.98**	0.07	0.38	**0.88**
Country would be better off	**0.99**	0.25	**0.76**	**0.68**
God in the Bible	**0.99**	0.01	**0.89**	0.05
Adam and Eve	**0.87**	0.07	**0.72**	0.27
Latent class probabilities	0.36	0.25	0.23	0.16
1997 survey (*n* = 498)				
Religious attendance	**0.76**	0.01	0.11	0.46
Religious intensity	**0.99**	0.21	**0.59**	**0.74**
Country would be better off	**0.97**	0.17	**0.91**	**0.70**
God in the Bible	**0.98**	0.04	**0.97**	0.08
Adam and Eve	**0.83**	0.12	**0.78**	0.25
Latent class probabilities	0.38	0.30	0.17	0.15

Bold indicates item response probabilities over 0.5

We labeled the four latent classes as follows: (1) *strongly religious* (item response probabilities in all indicators were over 0.5; 42% in 1971; 36% in 1988; 38% in 1997, (2) *privately religious* (item response probabilities in all indicators were over 0.5 excepting religious membership and participation; 28% in 1971; 23% in 1988; 17% in 1997), (3) *weakly religious* (item response probabilities in all indicators were under 0.5; 17% in 1971; 25% in 1988; 30% in 1997), and (4) *liberally religious* (item response probabilities in religious membership, participation, intensity, and civic value of religion were over 0.5 but two literal beliefs were under 0.5; 13% in 1971; 16% in 1988; 15% in 1997).

### Cox Proportional Hazard Models

Relative risk ratios from the Cox proportional hazard models are displayed in Table 4. Our second hypothesis is supported for males in Model 1. The mortality risk of weakly religious (*β* = 1.53, *p* < 0.05) and privately religious (*β* = 1.61, *p* < 0.05) males was 50–60%% higher compared to strongly religious males. There was no significant difference for liberally religious males.

**Table 4:** Cox models predicting time to death from baseline, with 1971, 1988, and 1996 religiosity and controls, longitudinal study of generations (*N* = 675)

1971 demographics	Males (*n* = 305)		Females (*n* = 370)	
	Model 1	Model 2	Model 1	Model 2
Age in 1971	1.07***	1.06***	1.06***	1.06***
Education in 1971 (reference = less than high school)				
High school or above	0.84	1.07	0.92	1.10
Some college	0.62*	0.80	1.00	1.33
Bachelor’s degree or above	0.47***	0.71	0.79	0.95
Married (time-varying)	0.73 +	0.60**	0.99	0.91
1971 religious denomination (reference = mainline protestant)				
Evangelical protestant	1.11	1.22	1.15	1.00
Catholic	1.19	1.00	0.93	0.81
Jewish	0.85	0.91	1.00	0.86
Others	1.22	1.06	0.77	0.79
None	1.49	1.08	1.11	0.99
Religiosity continuous membership probability (reference = strongly religious)				
Weakly religious	1.53*	1.32	1.44	1.00
Privately religious	1.61*	1.49*	1.22	0.91
Liberally religious	1.29	0.87	1.52 +	1.29
Potential mediators (time-varying)				
Self-rated health		0.20***		0.26***
Missingness in self-rated health		1.87***		2.12***
Bradburn psychological wellbeing		1.04		1.00

+ *p* < 0.1; *p < 0.05; **p < 0.01; ***p < 0.001

Results among the females in our sample only partially supported this hypothesis—there was a marginally significant association between mortality and the probability of being liberally religious in 1971, 1988, or 1997 (*β* = 1.52, *p* < 0.10), but no association with the probability of being weakly or privately religious. Having a specific religious denomination in 1971 was not associated with mortality risk after 1979.

Other than confirming previous research regarding the relationship between self-rated health in midlife and mortality risk in later life, our third hypothesis was only partially supported. The addition of the time-varying control for self-rated health in Model 2 did mediate the association between religiosity and mortality risk for privately and weakly religious males and liberally religious females; however, the addition of psychological wellbeing was not associated with mortality risk, nor did it mediate any of the associations with mortality risk in the model.

Among males in our sample, self-rated health (*b* = 0.19, *p* < 0.001) and missing on self-rated health (*b*= 1.75, *p* < 0.001) were significant and reduced the association between mortality risk and the probability of being privately religious (decreased from *b* = 1.61, *p* < 0.05 to b = 1.49, *p* < 0.10) or weakly religious (*b* = 1.32, *p* > 0.10) compared to the probability of being strongly religious. Among females in our sample, self-rated health (*β* = 0.26, *p* < 0.001) and missing on self-rated health (*β* = 2.12, *p* < 0.001) fully explained the association between mortality risk and the probability of being liberally religious (decreased from *β* = 1.52, *p* < 0.10 to *β* = 1.29, *p* > 0.10) in comparison with the probability of being strongly religious.

Each additional year of age at baseline was associated with a 6% increase in mortality risk for men (*β* = 1.06, *p* < 0.001) and women (*β* = 1.06, *p* < 0.001) in the fully specified model. Among other controls in the fully specified model (Model 2), being married over time (*β* = 0.60, *p* < 0.01) was associated with lower risk of mortality for males only, and being in better health was associated with lower risk of mortality for males (*β* = 0.20, *p* < 0.001) and females (*β* = 0.26, *p* < 0.001).

## Discussion

The current study found associations between religiosity and mortality in male or female G2 adults, when controlling for sociodemographic factors and including mediators for health and psychological wellbeing, between religiosity in 1971, 1988, and 1997 and mortality risk between 1979 and 2020 in the LSOG sample.

Results confirmed the first hypothesis. We found four distinct classes of religiosity in all three waves in this sample of the pre-baby boomer cohort: strongly religious, weakly religious, liberally religious, and privately religious. We demonstrated diversity in how religious practice, beliefs, and identity are configured with consistently strong and consistently weak classes, with other classes heterogenous with respect to their standing on those dimensions. Further, in earlier research we identified similar latent classes of religiosity among Baby Boomers and Generation X (Hwang et al., 2024, 2023a, 2023b), improving confidence in the validity of our current findings.

Results partially supported our second hypotheses, that those who were strongly religious at baseline had lower risk of mortality compared to those who were less religious. In this sample, being weakly or privately religious for males and being liberally religious for females was associated with greater risk of mortality over the subsequent five decades relative to the strongly religious. The greater risk among males who scored lower on religious participation should not be surprising in light of the social control and social integration functions of religion (McCullough et al., 2000) that may primarily benefit older males. In contrast, the higher level of religious participation does not appear protective for liberally religious females; this could be because older females more likely to derive such benefits from their personal networks than from religious services; but this does not explain why strongly religious females have lower mortality risk. Unfortunately, our analysis did not include a control for social connectedness or social activities outside of religion that might have helped to clarify this difference between females in the strongly and liberally religious classes.

Results also partially supported our third hypotheses that accounting for health and psychological wellbeing will partially explain the association between religiosity and mortality found in our second hypothesis. In this sample, self-rated health over time was a predictor of lower mortality risk for males and females, but psychological wellbeing was not. Furthermore, self-rated health partially explained the elevated mortality risk for privately religious males, and fully explained the elevated mortality risk for weakly religious males and liberally religious females. We can speculate that self-rated health is a stronger influence on mortality risk in males who are less involved in religious participation, given the aforementioned social benefits to older males, but we do not know why identifying as less religious or not embracing literal beliefs leads to self-rated health having a stronger effect on mortality. Similarly, liberally religious females may be more impacted by self-rated health because poor health impairs other social activities or connections, which we do not measure in this study; but again, without these additional measures, we cannot know why self-rated health has a stronger influence on mortality in liberally religious females than in strongly religious females.

### Limitations

This study had several limitations. Most notable is the sample’s geographic concentration in the Southern California region and its relative lack of ethnic and racial diversity. Thus, we urge caution in generalizing our findings beyond the regional and background confines of the study population. Additionally, our models do not include measures of social networks or social activities outside of religion, which limits our ability to fully explain what other factors may be impacting the associations explored in this study. Finally, we relied on a fairly narrow selection of religion measures that omitted typical questions about frequency of prayer, belief in God, and spiritual practices, and contained questions that are biased toward Judeo-Christian beliefs. Therefore, caution is urged in accepting our typological model as representative across all religious contexts.

A perennial question raised in connection with interpreting observed associations between religion and mortality outcomes is whether results are truly causal or whether less healthy individuals shy away from engaging in religious activities (Levin, 1994). Our measures of religiosity were taken from several periods during midlife and early old age, but mostly prior to the stage of life when frailty substantially impacts religious participation. One study using LSOG data found that religious service attendance was stable through most of adult life and did not begin to decline until about 80 years of age (Hayward & Krause, 2013b). Thus, the timing of our measurements of religiosity discounts the possibility that findings were due to endogenous processes whereby those with poor health reduced their religious involvement.

In sensitivity tests (not reported), restricting the measures of religion to the baseline period only—when the sample was almost uniformly in good health—also showed that strongly religious men had a longevity advantage, which did not alter the overall conclusions of the research. Restricting the religiosity measures to baseline also resulted in self-rated health having a mediation effect on mortality risk among the weakly and privately religious men. Therefore, restricting religiosity to baseline only oversimplified the relationship between religiosity, health, and mortality among males in this sample, but ultimately did not dramatically alter the results.

### Implications for Research

We suggest that alternative models be tested to confirm that the association between religiosity and mortality has a causal basis, such as the use of sibling fixed effects models which controls shared family environment and genetic sources of frailty when assessing underlying mortality risk. Finally, the pathways by which religiosity leads to lower mortality risk requires attention to potential mediators of risk related to social, psychological, and spiritual resources available to religious individuals, as well as the tendency to engage in healthful behaviors and avoid risky behaviors. Unfortunately, these potential mediators were inconsistently measured across waves of the LSOG.

We suggest that future research delve into the implications of religious transitions for mortality risk. While prior research has found substantial intergenerational stability in strongly and weakly religious types over several decades, heterogeneous religious patterns have been found to be more in flux (Hwang et al., 2021). Stability and change in religiosity have consequences for long-term psychological wellbeing in midlife and beyond (Hwang, et al., 2022) and the same may apply to physical wellbeing.

## Conclusion

Similar to previous studies, our findings on the association between religiosity and mortality differ based on gender. Our results endorse the perspective that men derive greater benefit from religious involvement in terms of mortality, potentially because of the social integration function of religious participation, while health and psychological wellbeing are more influential than religious participation in terms of women’s mortality. Our analyses have highlighted the utility of using a typological approach in studying the survival benefits of religiosity. This typological approach accounts for multiple dimensions of religiosity, rather than relying on oversimplified understandings of religiosity as merely religious practices or religious beliefs, as well as the intersection among these dimensions. Such an approach can more inclusively represent the complexity of religious and quasi-religious orientations and include a diversity of profiles such as those who are spiritual-but-not-religious and those who practice non-Western religious traditions. Our research is a first step toward bringing together recent scholarship using a multidimensional person-centered framework to represent religion with the rich scholarship on the role that religion plays in promoting health and wellbeing over the second half of life.

## Appendix 1. General Health Items, Longitudinal Study of Generations, 1971

**Table:**

Introduction: Please check “yes” beside an item that describes your general health. Please check “no” beside an item that does not describe your situation. Please check “?” if that is how you feel, or if the question does not apply to you		
Item	Question	Coding
HEALTH1	Have a lot of minor ailments	Yes = 0, No = 1
HEALTH2	Need little or no medical care	Yes = 1, No = 0
HEALTH3	Feel tired all the time	Yes = 0, No = 1
HEALTH4	Must be careful what I do	Yes = 0, No = 1
HEALTH5	Excellent	Yes = 1, No = 0
HEALTH6	Failing	Yes = 0, No = 1
HEALTH7	Never felt better	Yes = 1, No = 0
HEALTH8	Poor	Yes = 0, No = 1
HEALTH9	Better condition than most people my age	Yes = 1, No = 0

## Appendix 2. Bradburn Affect Balance Scale Items, Longitudinal Study of Generations, 1971, 1988, and 1997

**Table:**

Questions: during the past few weeks, did you ever feel–	Coding
Particularly excited or interested in something?	Yes = 1, No = 0
So restless that you couldn’t sit long in a chair?	Yes = 0, No = 1
Proud because someone complimented you on something you had done?	Yes = 1, No = 0
Very lonely or remote from other people?	Yes = 0, No = 1
Pleased about having accomplished something?	Yes = 1, No = 0
Bored?	Yes = 0, No = 1
On top of the world?	Yes = 1, No = 0
Depressed or very unhappy?	Yes = 0, No = 1
Felt that things were really going your way?	Yes = 1, No = 0
Upset because someone criticized you?	Yes = 0, No = 1
